# Arg/Lys-containing IDRs are cryptic binding domains for ATP and nucleic acids that interplay to modulate LLPS

**DOI:** 10.1038/s42003-022-04293-w

**Published:** 2022-12-01

**Authors:** Mei Dang, Tongyang Li, Shibo Zhou, Jianxing Song

**Affiliations:** Department of Biological Sciences, Faculty of Science, National University of ingapore, 10 Kent Ridge Crescent, Singapore, 119260 Singapore

**Keywords:** Intrinsically disordered proteins, Solution-state NMR, Solution-state NMR

## Abstract

Most membrane-less organelles (MLOs) formed by LLPS contain both nucleic acids and IDR-rich proteins. Currently while IDRs are well-recognized to drive LLPS, nucleic acids are thought to exert non-specific electrostatic/salt effects. TDP-43 functions by binding RNA/ssDNA and its LLPS was characterized without nucleic acids to be driven mainly by PLD-oligomerization, which may further transit into aggregation characteristic of various neurodegenerative diseases. Here by NMR, we discovered unexpectedly for TDP-43 PLD: 1) ssDNAs drive and then dissolve LLPS by multivalently and specifically binding Arg/Lys. 2) LLPS is driven by nucleic-acid-binding coupled with PLD-oligomerization. 3) ATP and nucleic acids universally interplay in modulating LLPS by competing for binding Arg/Lys. However, the unique hydrophobic region within PLD renders LLPS to exaggerate into aggregation. The study not only unveils the first residue-resolution mechanism of the nucleic-acid-driven LLPS of TDP-43 PLD, but also decodes a general principle that not just TDP-43 PLD, all Arg/Lys-containing IDRs are cryptic nucleic-acid-binding domains that may phase separate upon binding nucleic acids. Strikingly, ATP shares a common mechanism with nucleic acids in binding IDRs, thus emerging as a universal mediator for interactions between IDRs and nucleic acids, which may underlie previously-unrecognized roles of ATP at mM in physiology and pathology.

## Abstract (web summary)

Probing ssDNA binding to TAR-DNA-binding protein-43 (TDP-43) prion-like domain (PLD) through NMR shows key interactions for nucleic acid to drive liquid-liquid phase separation (LLPS) of intrinsically-disordered proteins.

## Introduction

Liquid–liquid phase separation (LLPS) has been recently established as the common principle for forming membrane-less organelles (MLOs), which include nucleoli, Cajal bodies, nuclear speckles, paraspeckles, histone-locus bodies, nuclear gems, and promyelocytic leukemia (PML) bodies in the nucleus as well as P-bodies, stress granules, and germ granules in the cytoplasm^1–5^. Intriguingly, most, if not all, MLOs are composed of both nucleic acids and proteins rich in intrinsically disordered regions (IDRs). Currently, while IDRs have been well-recognized to drive LLPS, the effect and mechanism of nucleic acids to affect LLPS remain largely elusive. In particular, one fundamental question remaining to be answered is whether nucleic acids affect LLPS just by non-specific electrostatic/salt effects or by specific binding. As a consequence, the high-resolution mechanism is completely absent for nucleic acids to interact with IDRs in LLPS.

TAR-DNA-binding protein-43 (TDP-43) was originally identified to be a human protein capable of binding the trans-active response (TAR) DNA region of HIV to repress its transcription^6^. Now TDP-43 has been uncovered to be a nuclear protein that functions by binding a large array of RNA and single-stranded DNA (ssDNA) including more than 6000 RNA species of diverse sequences^7,8^. Furthermore, in response to stresses, TDP-43 also becomes localized into the cytoplasm to participate in forming stress granules (SGs) with RNAs through phase separation. Most intriguingly, under pathological conditions, TDP-43 accumulates and forms inclusion bodies in the cytoplasm of motor neurons, which is a common pathological hallmark of most cases of amyotrophic lateral sclerosis (ALS), as well as associated with an increasing spectrum of other major neurodegenerative diseases, including Alzheimer’s (AD), Parkinson’s (PD), frontotemporal dementia (FTD), and Huntington’s (HD) diseases^7–14^.

The 414-residue TDP-43 is composed of the folded N-terminal domain (NTD)^15,16^ and two RNA recognition motif (RRM) domains for binding various RNA and DNA^17–20^, as well as the C-terminal low-complexity (LC) domain over the residues 265–414 with the amino acid composition similar to those of yeast prion proteins, thus designated as the prion-like domain (PLD)^14,21,22^. Puzzlingly, although TDP-43 PLD is intrinsically disordered, it hosts almost all ALS-causing mutations identified so far^7–9^. Intriguingly, as compared with PLDs in other RRM-containing proteins, TDP-43 PLD is unique in owning a hydrophobic region over residues 311–343, which adopts the partially folded helical conformation in solution^21,22^. Recently, TDP-43 PLD has been characterized to critically drive LLPS of TDP-43, which is essential for forming SGs but might further exaggerate into pathological aggregates or amyloid fibrils^21–31^. So far, LLPS of TDP-43 PLD has been extensively characterized without nucleic acids to be driven mainly by the formation of the dimeric/oligomeric helix over the unique hydrophobic region^22,25,26^, which appears also to undergo conformational exchanges with the amyloid-like β-rich oligomers^30^.

Nevertheless, so far it remains unknown how nucleic acids modulate LLPS of TDP-43 PLD, non-specifically or specifically? Although cellular environments where TDP-43 plays its physiological and pathological roles are rich in various nucleic acids and LLPS of TDP-43 has been indeed found to be affected by both RNA^32^ and ssDNA^33^. Furthermore, ATP, the universal energy currency mysteriously with concentrations (>mM) much higher than required for its classic functions in all living cells^34^, was shown to act as a biological hydrotrope to dissolve LLPS of RRM-containing proteins including TDP-43 at >5 mM^35,36^. Very recently, we found that ATP is capable of biphasically modulating LLPS of TDP-43 PLD: namely induction at low but dissolution at high ATP concentrations^37^. Most unexpectedly, despite containing only five Arg and one Lys residue within the 150-residue PLD: namely Arg268, Arg272, Arg275, Arg293, and Arg361, as well as Lys408, our residue-specific NMR results unambiguously indicate that ATP achieves the biphasic modulation of LLPS by acting as a bivalent binder to specific binding to Arg/Lys residues through electrostatic interactions between ATP triphosphate group and Arg/Lys side chain cations as well as π–π/π–cation interactions between ATP purine ring and Arg/Lys side chains^37^. Interestingly, the binding affinity of ATP to Arg was experimentally shown to be much higher than that to Lys^37–39^.

Here as assisted by turbidity measurement and DIC imaging, we utilized NMR spectroscopy to visualize the effects on LLPS and binding events of Tar32, a natural ssDNA ligand of TDP-43 together with two non-specific ssDNAs A6 and A32 with the wild-type (WT) and two mutated PLDs: namely Del-PLD with residues 311–343 deleted and AllK-PLD with all five Arg replaced by Lys residues^37^. Although ssDNA and RNA only have two minor differences in chemical structures: the base thymine (T) in DNA is replaced by uracil (U) in RNA, while d-2-deoxyribose in DNA is replaced by d-ribose in RNA, unlike RNA which is vulnerable to degradation by RNAse extensively existing in environments, ssDNA has very high chemical stability, which thus allows acquiring time-consuming NMR spectra.

The results decode: (1) all three ssDNAs could drive and then dissolve LLPS of TDP-43 WT-PLD with the capacity dependent on the length but not sequence. Most unexpectedly, ssDNAs modulate LLPS by specifically binding Arg/Lys with an affinity to Arg higher than that to Lys. (2) In the presence of nucleic acids, LLPS of TDP-43 PLD is driven by the nucleic acid-binding coupled with PLD-oligomerization. Nevertheless, in the general context, the multivalent nucleic acid-binding itself is sufficient to drive LLPS of Arg/Lys-containing IDRs without the need for any other driving force. (3) ATP and ssDNAs interplay in modulating LLPS by competing for binding Arg/Lys. However, for TDP-43 PLD, the presence of the unique hydrophobic region renders its LLPS to be prone to exaggerating into aggregation. The study unveils the first residue-resolution mechanism of the nucleic-acid-driven LLPS of TDP-43 PLD, which emphasizes the specific role of the multivalent nucleic-acid-binding in driving LLPS as well as the unique potential of the hydrophobic region to exaggerate functional LLPS into pathological aggregation. Remarkably, a general principle is emerging that not just TDP-43 PLD, but all Arg/Lys-containing IDRs, which account for a large portion of eukaryotic proteomes, are cryptic nucleic-acid-binding domains and may undergo LLPS upon multivalently binding nucleic acids. Most strikingly, ATP shares a common mechanism with nucleic acids in binding Arg/Lys residues of IDRs, thus emerging as a general mediator for interactions between IDRs and nucleic acids. This may represent a key mechanism underlying previously unrecognized roles of ATP at mM in physiology and pathology.

## Results

### ssDNAs biphasically modulate LLPS with the length-dependent capacity

**Fig. 1:**
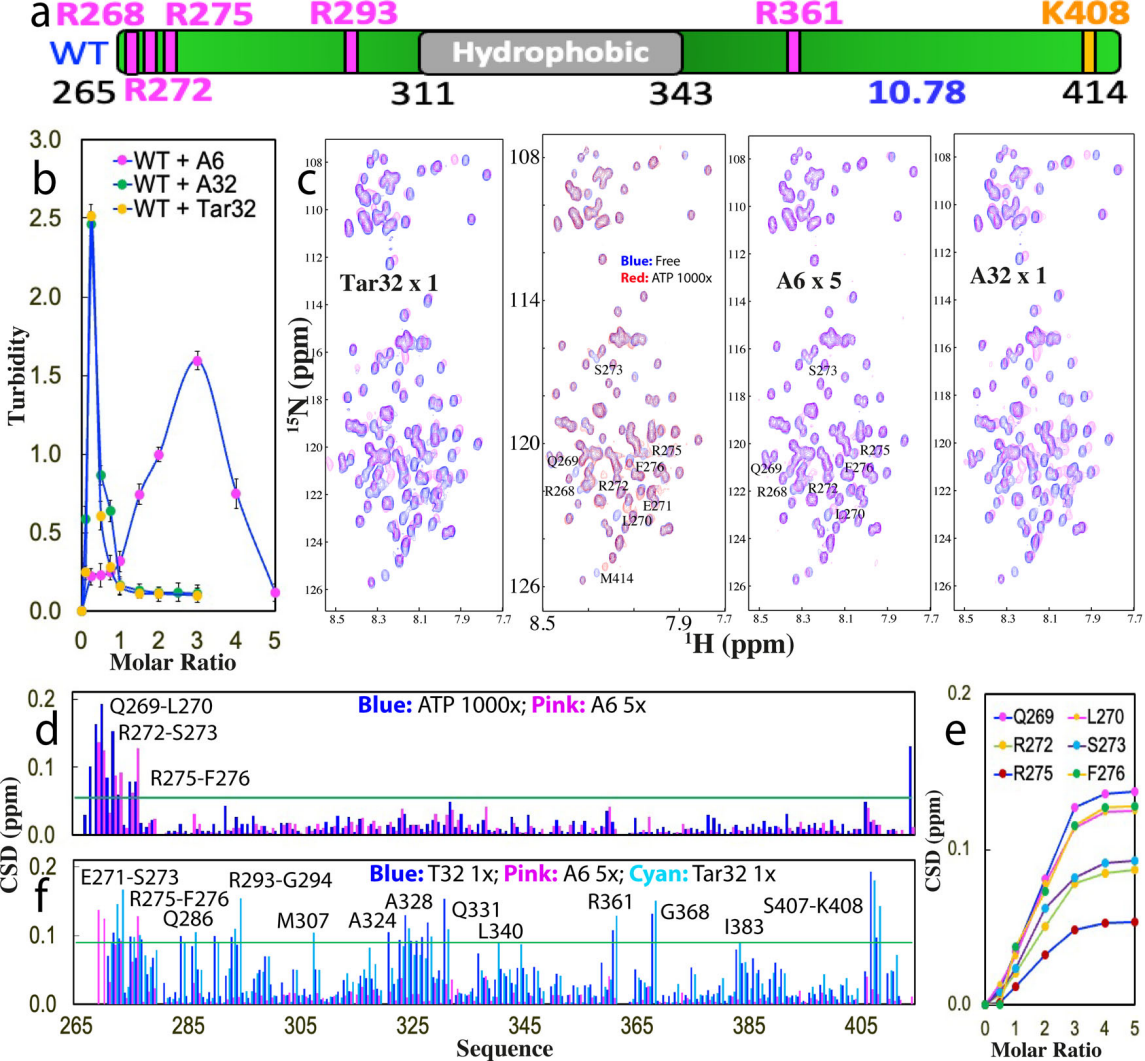
Three ssDNAs length-dependently modulate LLPS of TDP-43 WT-PLD. **a** Schematic representation of TDP-43 WT-PLD with five Arg and one Lys, as well as p*I* value indicated. **b** Turbidity curves from three repeated measurements (*n* = 3) of WT-PLD in the presence of Tar32, A6, and A32 at different molar ratios. **c** ^1^H–^15^N NMR HSQC spectra of the ^15^N-labeled WT-PLD in the absence (blue) and in the presence of ATP at a molar ratio of 1:1000 (red), as well as A6 at 1:5, A32 and Tar32, respectively, at 1:1 (purple). **d** Chemical shift difference (CSD) of WT-PLD between the free state and in the presence of ATP at 1:1000 (blue) or A6 at 1:5 (purple), respectively. The green line is used to indicate the value (0.05): Average + STD at the ratio of 1:5. The residues with CSD values > Average + SD are defined as significantly perturbed residues and labeled. **e** CSD tracings of six significantly perturbed residues in the presence of A6 at different ratios. **f** Chemical shift difference (CSD) of WT-PLD between the free state and in the presence A6 at 1:5 (purple), A32 at 1:1 (blue), and Tar32 at 1:1 (cyan), respectively. The green line is used to indicate the value (0.09): Average + STD in the presence of Tar32 at the ratio of 1:1 (PLD: Tar32). The residues with CSD values > Average + SD are defined as significantly perturbed residues and labeled.

Previously, by NMR we have characterized the solution conformation and amyloid fibrillation^21^ as well as its interaction with ATP^37^ for the 150-residue TDP-43 PLD over residue 265–414 with a p*I* of 10.78 (Fig. 1a). Here by measurement of turbidity (absorption at 600 nm) and DIC imaging at the same protein concentrations (15 μM) and in the same buffer which we previously used^37^, we first assessed how LLPS of TDP-43 WT PLD is affected by a 32-mer ssDNA Tar32, a natural ligand of TDP-43, whose sequence is derived from the trans-active response (TAR) DNA of HIV^6,33^, and contains all four nitrogenous bases (Supplementary Fig. 1).

Strikingly, upon stepwise addition of Tar32, the turbidity of TDP-43 PLD rapidly increased with a value of 0.25 at a molar ratio of 1:0.1 (PLD:Tar32), and then reached the highest value of 2.51 at 1:0.25 (Fig. 1b). DIC imaging showed that many liquid droplets were formed, some of which have diameters of ~2 μm (Supplementary Fig. 2). Intriguingly, further addition of Tar32 led to the reduction of turbidity and at 1:1, the turbidity value was only ~0.11, where no droplet could be detected by DIC imaging. The results clearly indicate that Tar32 is able to biphasically modulate LLPS of TDP-43 WT PLD: namely to drive LLPS at low Tar32 concentrations but dissolve at high concentrations.

To assess whether the unique sequence of Tar32 is essential for modulating LLPS, we subsequently used a 32-mer ssDNA A32 containing only adenine (A) (Supplementary Fig. 1a) to titrate WT PLD under the same conditions. As shown in Fig. 1b, the turbidity curve of A32 is very similar to that of Tar32 with the highest value at 1:0.25. DIC visualization indicates that A32 also drove and then dissolved the formation of droplets with an overall pattern very similar to that of Tar32. Therefore, this set of results suggests that the specific sequence of Tar32 is not essential for biphasically modulating LLPS.

Next, we set to test whether the length of ssDNA is critical by titrating WT-PLD with a 6-mer ssDNA A6. As shown in Fig. 1b, for A6, the turbidity reached the highest (1.59) at 1:3 and at 1:5 the turbidity reduced to 0.12 (Fig. 1b). DIC imaging indicates that A6 did drive and then dissolve the formation of the droplets, but the number induced by A6 at 1:3 is less than that by Tar32 or A32 at 1:0.25, thus resulting in a smaller value. The results with Tar32, A32, and A6 together suggest that it is the length of ssDNA but not the sequence that is the key determinant for the capacity of ssDNA in biphasically modulating LLPS of TDP-43 PLD.

### NMR visualization of the biphasic modulation of LLPS by ssDNAs

To insight into the binding events for ssDNAs to drive and subsequently dissolve LLPS of TDP-43 WT-PLD, under exactly the same conditions as above for turbidity measurement and DIC imaging, we monitored the titrations of Tar32, A6, and A32 into WT-PLD by NMR HSQC spectroscopy, which is particularly powerful in pinpointing the binding events with a wide range of affinity at a residue-specific resolution^38–42^.

Interestingly, for Tar32, its addition at ratios <0.25 triggered no large shift of HSQC peaks of WT PLD (Supplementary Fig. 3). However, at the ratio of 1:0.25 where the turbidity has the highest value and many droplets were formed, a large set of HSQC peaks became too broad to be detected, which appeared to result from the formation of large and dynamic Tar32-PLD complexes whose HSQC peaks became too broad to be detected. Nevertheless, with further addition of Tar32, most disappeared peaks were restored at 1:0.5 and no large shift was observed with the ratio further increased to 1:1, where all droplets were completely dissolved. Strikingly, at 1:1, a large set of HSQC peaks were significantly shifted as compared with those in the free state (Fig. 1c), indicating that a large set of residues were perturbed by Tar32.

We then titrated WT PLD with A6 under the same conditions. Briefly, A6 induced the broadening of HSQC peaks at 1:3 where the turbidity value reached the highest and many droplets were formed. The intensity of most broadened peaks became largely restored and the shift of HSQC peaks was mostly saturated at 1:5, where all droplets were dissolved (Supplementary Fig. 4). Different from what was observed on Tar32, the addition of A6 only triggered the shift of a small set of HSQC peaks (Fig. 1c), whose pattern is very similar to that induced by ATP as we previously observed^37^. Moreover, the titrations of A32 led to changes in HSQC peaks with an overall pattern very similar to that of Tar32 (Supplementary Fig. 5).

Detailed NMR assignments revealed that for A6, except for the residues Arg268 which had a significant shift by ATP but became too broad to be detectable by A6, as well as Met414 which had a significant shift by ATP but no large shift by A6, the residues with significantly shifted HSQC peaks are very similar to those induced by ATP, which are all clustered over N-terminal three Arg residues including Gln269-L270, Arg272-Ser273, and Arg275-Phe276 (Fig. 1d). Strikingly, the peaks from these residues gradually shifted upon stepwise addition of A6 and the shift was largely saturated at 1:5 (Fig. 1e). This indicates that the binding affinity of A6 is relatively weak and in the fast exchange regime of NMR chemical shift time scale^40^.

By contrast, the residues significantly perturbed by Tar32 and A32 are much more profound and located over the whole sequence which includes Glu271-Ser273, Arg275-Phe276, Gln286, Arg293-Gly294, Met307, Ala324, Ala328, Gln331, Leu340, Arg361, Gly368, Ile383, Ser407-Lys408 (Fig. 1f). Overall, the shift patterns induced by Tar32 and A32 are very similar although the amplitude of shifts is different to some degree (Fig. 1f). The results suggest that in binding with TDP-43 PLD: (1) Tar32 and A32 have the binding affinity much higher than that of A6, and thus their affinity is in the slow exchange regime of the NMR chemical shift time scale^40^; (2) Tar32 and A32 have the highly similar binding sites and affinity. As Tar32 and A32 have the same backbone but different bases, the NMR chemical shift changes together with the above turbidity and DIC results imply that different bases have a highly similar binding affinity to Arg/Lys residues.

The current results together with the previous ones with ATP^37^ reveal that ATP, A6, Tar32, and A32 appear to bind Arg/Lys residues of TDP-43 PLD with a common mechanism as they are all composed of the same building unit: nucleotide. As such, they are all able to establish electrostatic interactions between the phosphate group of nucleotide and side chain cations of Arg/Lys as well as π–π/π–cation interactions between base aromatic rings and Arg/Lys side chains. The results that Tar32 containing all four bases and A32 consisting of only adenine have highly similar modulating capacity suggest that four bases, regardless of adenine (A)/guanine (G) with a purine aromatic ring, or thymine (T)/cytosine (C) with a pyrimidine aromatic ring, all have a highly similar affinity in establishing π–π/π–cation interactions with side chains of Arg/Lys residues of IDRs.

Nevertheless, as ATP, A6, Tar32/A32 have different numbers of covalently linked nucleotides, they are expected to have length-dependent affinities, because it is well known that for a multivalent binder, its dissociation constant (*K*_d_) value is the time of *K*_d_ values of the individual binding events if assuming these binding events are independent^43^. As such, ATP can only establish bivalent binding and therefore has a low affinity to Arg/Lys. On the other hand, A6 and A32/Tar32 have multiple covalently-linked nucleotides and thus can establish a multivalent binding. In particular, as A32/Tar32 is much longer than A6 and consequently one A6 molecule is only able to maximally achieve multivalent binding to the clustered Arg268, Arg272, and Arg275 but one Tar32/A32 molecule is able to bind most, if not all, Arg/Lys residues of TDP-43 PLD as evidenced by the NMR results that Tar32/A32 could perturb residues over the whole PLD molecule. As a result, Tar32/A32 binds PLD with an affinity much higher than that of A6. The perturbations to residues other than Arg/Lys residues by Tar32/A32 might not be due to the direct binding with A32/Tar32, but result from the avoidable close contacts of these residues with A32/Tar32, or/and conformational/dynamic changes of these residues induced by the binding of A32/Tar32 to Arg/Lys, as we previously observed on the binding of ATP with TDP-43 PLD^37^. Indeed, previously we showed that ssDNAs showed no detectable binding to the 165-residue PLD of FUS, which is completely absent of Arg/Lys^38^.

### ssDNAs bind Arg and Lys residues with distinctive affinities

**Fig. 2:**
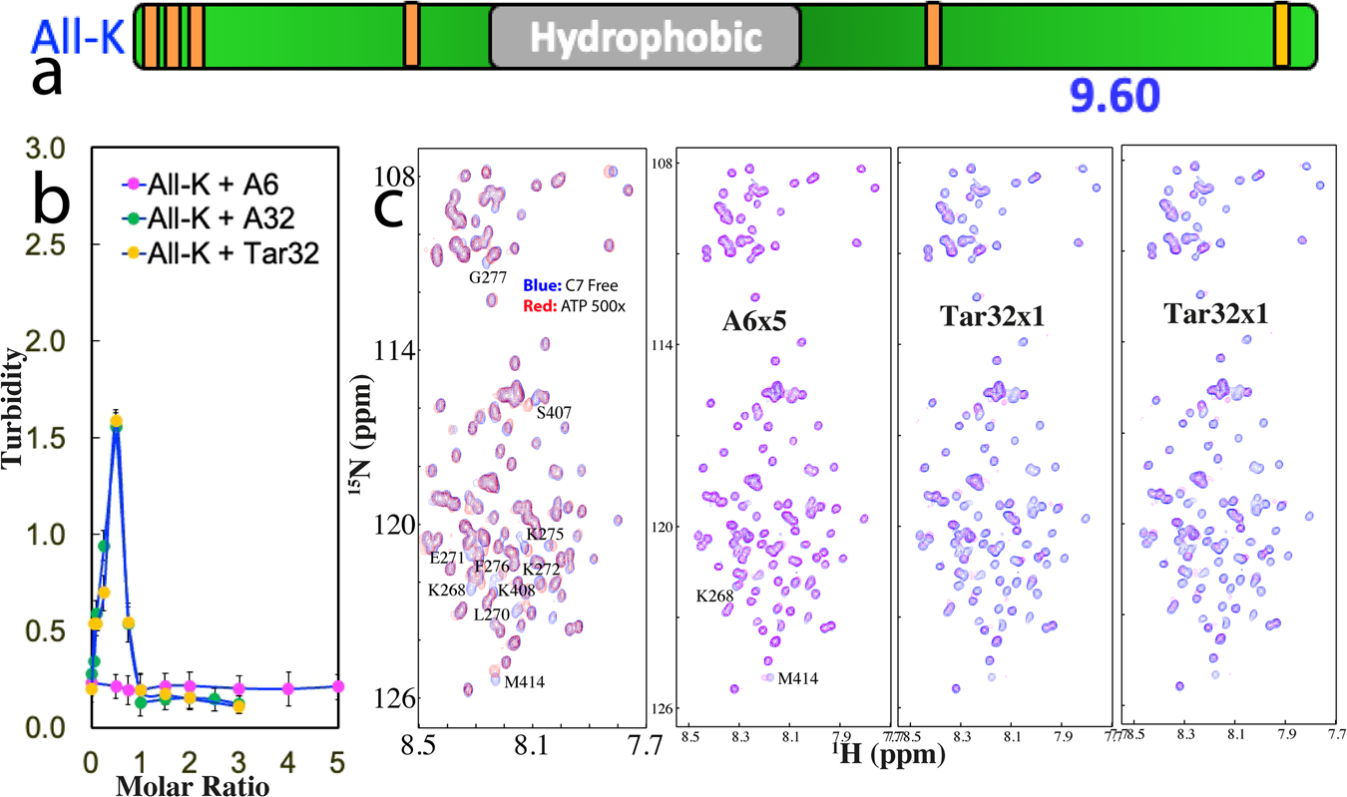
Three ssDNAs bind to Arg with the affinity higher than that to Lys. **a** Schematic representation of AllK-PLD with all five Arg mutated to Lys. **b** Turbidity curves of AllK-PLD in the presence of Tar32, A6 and A32 at different molar ratios. **c** HSQC spectra of AllK-PLD in the absence (blue) and in the presence of ATP at a molar ratio of 1:500 (red), as well as A6 at 1:5, A32 and Tar32 at 1:1 (purple) respectively.

Previously we found that the binding affinity of ATP to Arg is much higher than that to Lys^37^. Here we addressed the question of whether this observation also holds for ssDNAs by titrating A6, Tar32, and A32 into AllK-PLD with its p*I* (9.6) only slightly lower than that of WT-PLD (Fig. 2a), which we previously constructed and characterized by replacing all five Arg residues with Lys^37^. Intriguingly, previously we found that Arg residues in fact behave to conformation-specifically inhibit LLPS of TDP-43 PLD and consequently AllK-PLD could weakly undergo LLPS at 15 μM even in the free state, with a turbidity of 0.23 (Fig. 2b) and formation of some small droplets with diameters of ~0.7 μm (Supplementary Fig. 6).

We then titrated A6 into AllK-PLD but even up to 1:5, A6 triggered no large change of turbidity (Fig. 2b) as well as the number and size of droplets. By contrast, the addition of Tar32 increased the turbidity which reached the highest (1.59) at 1:0.5. DIC imaging showed that a large number of droplets were formed with some having diameter of ~1.5 μm. Further increase of Tar32 concentrations led to the reduction of the turbidity and number of droplets and at 1:1, the turbidity was only 0.15 and no droplet could be observed. Titrations of A32 resulted in a similar turbidity curve as well as induction and dissolution of droplets.

Interestingly, HSQC titrations showed that the addition of A6 had almost no large perturbation of HSQC peaks (Supplementary Fig. 7) and even at 1:5, only two peaks from Lys268 and Met414 showed small shifts (Fig. 2c), thus suggesting that the binding affinity of A6 to AllK-PLD is very weak. On the other hand, the addition of Tar32 triggered no large shifts of HSQC peaks of AllK-PLD at 1:0.1 but induced dramatic peak broadening at 1:0.5 (Supplementary Fig. 8), where the turbidity reached the highest. Further addition of Tar32 at 1:0.75 led to restoring some disappeared peaks but many HSQC peaks still remained too broad to be detectable. Intriguingly, at 1:1 where the droplets were completely dissolved, many peaks detectable at 1:0.75 became too broad to be detectable (Fig. 2c).

A very similar pattern of HSQC peak changes was observed on the titrations by A32 (Supplementary Fig. 9). The results imply that the binding affinity of Tar32/A32 to AllK-PLD is higher than that of A6 to WT-PLD but weaker than that of Tar32/A32 to WT-PLD. As such, the binding affinity of Tar32/A32 to AllK-PLD is in the intermediate exchange regime of the NMR chemical shift time scale characteristic of severe broadening of NMR peals, while that of A6 to WT-PLD is in the fast exchange regime and that of Tar32/A32 to WT-PLD is in the slow exchange regime.

Therefore, unlike A6 whose addition induced a gradual shift of HSQC peaks of WT-PLD and Tar32/A32 whose addition resulted in two sets of HSQC peaks of WT-PLD, the addition of Tar32/A32 into AllK-PLD even at an exceeding amount resulted in extensive broadening/disappearance of HSQC peak. The results together thus indicate that like what we observed with ATP^37^, the binding affinity of ssDNAs to Arg residues is also much higher than that to Lys, consistent with the previous observation on RNA^41^, because Arg side chain with a planar and delocalized guanidinium cation can establish both π–π and π–cation interactions with the base aromatic ring, while Lys side chain with a tetrahedral ammonium cation can only establish π–cation interactions with base aromatic ring (Supplementary Fig. 1d).

We have also attempted to assess the effect of ssDNAs on AllA-PLD with all Arg/Lys mutated to Ala, which became highly prone to aggregation^37^. Exactly as we previously observed with ATP^37^, the addition of ssDNAs also triggered an immediate precipitation of the sample and consequently, no further study could be performed.

### Nucleic-acid-driven mechanism of LLPS of TDP-43 PLD

**Fig. 3:**
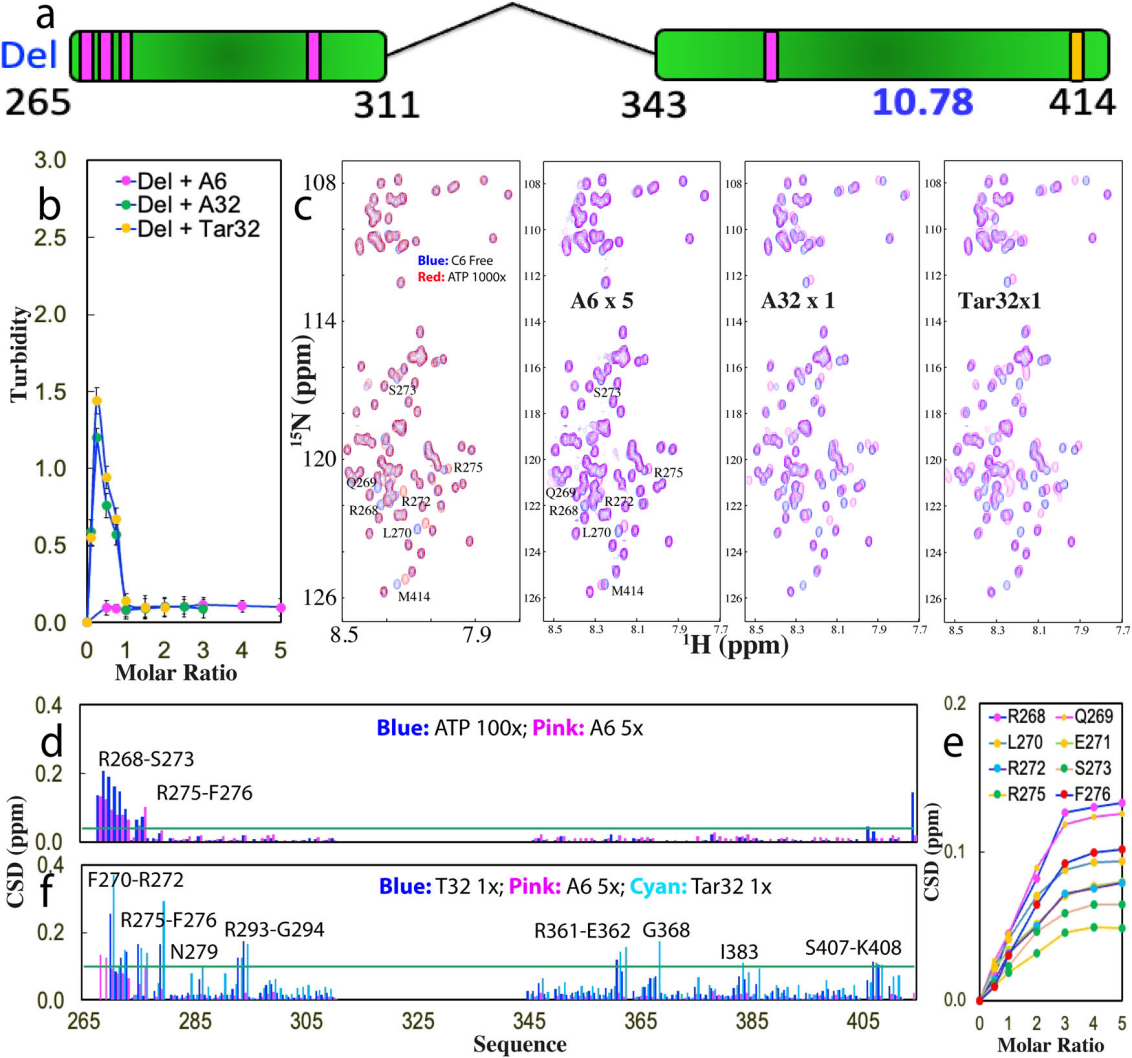
The hydrophobic region is not essential for LLPS driven by ssDNA. **a** Schematic representation of TDP-43 Del-PLD with residues 311-343 deleted. **b** Turbidity curves of Del-PLD in the presence of Tar32, A6 and A32 at different molar ratios. **c** HSQC spectra of Del-PLD in the absence (blue) and in the presence of ATP at a molar ratio of 1:1000 (red), as well as A6 at 1:5, A32 and Tar32 at 1:1 (purple). **d** Chemical shift difference (CSD) of Del-PLD between the free state and in the presence of ATP at 1:1000 (blue) and A6 at 1:5 (purple) respectively. The green line is used to indicate the value (0.04): Average + STD at the ratio of 1:5 (Del-PLD:A6). The residues with CSD values > Average + SD are defined as significantly perturbed residues and labeled. **e** CSD tracings of eight significantly perturbed residues in the presence of A6 at different ratios. **f** Chemical shift difference (CSD) of Del-PLD between the free state and in the presence A6 at 1:5 (purple), A32 at 1:1 (blue) and Tar32 at 1:1 (cyan) respectively. The green line is used to indicate the value (0.1): Average + STD in the presence Tar32 at the ratio of 1:1 (Del-PLD:Tar32). The residues with CSD values > Average + SD are defined as significantly perturbed residues and labeled.

The above results reveal that like ATP, three ssDNAs are able to biphasically modulate LLPS of RDP-43 WT-PLD by the specific binding to Arg/Lys. We then aimed to understand the nucleic-acid-driven mechanism of LLPS of TDP-43 PLD. Previously, in the absence of nucleic acids, the key driving force for LLPS of PLD has been extensively characterized to result from the dimerization/oligomerization of the hydrophobic region 311–343 uniquely existing in TDP-43 PLD. Furthermore, we have previously shown that for Del-PLD with the residues 311–343 deleted (Fig. 3a), ATP no longer induced its phase separation although it still binds the same set of residues with a highly similar affinity, indicating the intrinsic driving force is essential for ATP to induce LLPS^37^.

We first titrated Del-PLD with A6 but no large change was observed in the turbidity with the ratios even up to 1:5 (Fig. 3b). DIC imaging confirmed that no droplet was formed with the ratios up to 1:5. The results indicate that A6 was unable to induce LLPS of Del PLD. By contrast, upon titration by Tar32, the turbidity increased rapidly and reached the highest value (1.44) at 1:0.25 (Fig. 3b). DIC imaging showed that many droplets were observed with the diameters of some droplets reaching ~1.5 μm (Supplementary Fig. 10). Further addition led to the reduction of turbidity and at 1:1 the turbidity is only 0.14 where no droplets were detected by DIC imaging. Interestingly, the titrations with A32 resulted in patterns of turbidity and DIC changes very similar to those by Tar32.

We also characterized the interaction of Del-PLD with A6, Tar32, and A32 by NMR HSQC titrations (Supplementary Figs. 11–13). Interestingly, although A6 is incapable of inducing LLPS of Del-PLD, it could still trigger the shift of a small set of HSQC peaks very similar to that of Del-PLD induced by ATP, which include Arg268-Ser273 and Arg275-Phe276 (Fig. 3d). Similar to what was observed on WT-PLD titrated by A6 (Fig. 1e), HSQC peaks of Del-PLD residues also gradually shifted upon stepwise addition of A6 and the shift was largely saturated at 1:5 (Fig. 3e). By contrast, the residues significantly perturbed by Tar32 and A32 are much more profound which are over the whole sequence which includes Phe270-Arg272, Arg275-Phe276, Asn279, Arg293-Gly294, Arg361-Glu362, Gly368, Ile383, and Ser407-Lys408 (Fig. 3f).

The results indicate that despite the deletion of residues 311–343, the binding affinity of A6 to Del-PLD is very similar to that of WT-PLD and also in the fast exchange regime, while those of Tar32/A32 remain in the slow exchange regime. Therefore, the existence of the hydrophobic region 311–343 appears to have no detectable impact on the binding affinity of A6, A32, and Tar32 to Arg/Lys. Nevertheless, it has an important contribution to the strength of the driving force for LLPS. For A6 with the low binding affinity, although it still binds a similar set of residues of Del-PLD at similar affinity, it failed to induce LLPD because of the absence of the intrinsic driving force from the oligomerization of residues 311–343. Nevertheless, for Tar32/A32 with a strong binding affinity, their multivalent binding is sufficient to drive and then dissolve LLPS of Del-PLD even without the intrinsic driving force. So it becomes clear that although for the special case of TDP-43 WT-PLD, the intrinsic driving force is essential for driving LLPS in the absence of nucleic acids, a nucleic acid with sufficient length and binding affinity is sufficient to drive LLPS by multivalently and specifically binding Arg/Lys residues even with the intrinsic driving force completely deleted.

### ATP and ssDNAs interplay to modulate LLPS by competing for binding Arg/Lys

**Fig. 4:**
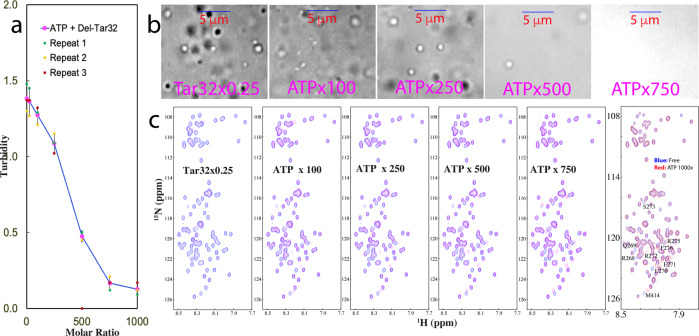
ATP dissolves LLPS of Del-PLD maintained by Tar32. **a** Turbidity curve from three repeated measurements (*n* = 3) of Del-PLD in the presence of Tar32 at 1:0.25, with further addition of ATP at different molar ratios. **b** DIC images of Del-PLD in the presence of Tar32 at 1:0.25, and with further addition of ATP at different molar ratios. **c** HSQC spectra of Del-PLD in the absence (blue) and in the presence of Tar32 at 1:0.25 (purple), as well as with further addition of ATP at different molar ratios.

As ATP and nucleic acids have been shown to specifically bind Arg/Lys residues, we thus set out to assess whether ATP and nucleic acids interplay in modulating LLPS of TDP-43 PLD by titrating ATP into four phases separated samples: namely WT-PLD in the presence of A6 at 1:3 (Supplementary Fig. 14); WT-PLD in the presence of Tar32 at 1:0.25 (Supplementary Fig. 15); AllK-PLD in the presence of Tar32 at 1:0.5 (Supplementary Fig. 16); and Del-PLD in the presence of Tar32 at 1:0.25 (Fig. 4).

Very unexpectedly, for WT-PLD in the presence of A6 at 1:3, the addition of ATP to 1:25 completely dissolved the liquid droplets as evidenced by the reduction of turbidity to 0.12, as well as dissolution of all droplets by DIC imaging. Strikingly, NMR HSQC titration indicates that upon the addition of ATP even at 1:25, the intensity of many broadened HSQC peaks due to phase separation in the presence of A6 becomes largely restored. Nevertheless, a number of peaks still remained to be very broad (Supplementary Fig. 14). Intriguingly, further addition of ATP to 1:50 rendered some restored peaks to become too broad to be undetectable and more peaks became undetected at 1:100. This may be due to the occurrence of dynamic aggregation induced by the non-specific screening effect of ATP and A6, both of which are highly negatively charged. Further addition of ATP above 1:100 resulted in complete precipitation of WT PLD and consequently, no NMR signals could be detected.

For WT PLD in the presence of Tar32 at 1:0.25, the addition of ATP up to 1:750 resulted in no large changes in turbidity and droplet number/size, implying that ATP exerts a minor effect on disrupting the Tar32–PLD complex which is jointly driven by both strong nucleic-acid-binding and intrinsic driving force. As monitored by NMR, the addition of ATP not only failed to restore the disappeared HSQC peaks, but rendered more peaks to become broad, and at 1:1000, most HSQC peaks became too broad to be detectable (Supplementary Fig. 15). This implies that the competition between ATP and Tar32 for binding Arg/Lys residues may shift the binding affinity of Tar32 to WT-PLD from the slow exchange regime to intermediate exchange regime, or dynamic aggregation occurred to some degree, both of which lead to a severe broadening of HSQC peaks. Intriguingly, further addition of ATP above 1:750 resulted in a complete precipitation and no NMR signal could be detected.

For AllK-PLD in the presence of Tar32 at 1:0.5, the addition of ATP up to 1:250 only resulted in a slight reduction in the number of the droplets (Supplementary Fig. 16a). On the other hand, as monitored by NMR, the addition of ATP to 1:50 led to the appearance of some peaks but further addition resulted in further disappearance of HSQC peaks. Upon addition of ATP above 1:250, the sample was completely precipitated and consequently, no NMR signal could be detected.

Very interestingly, for Del-PLD in the presence of Tar32 at 1:0.25, the addition of ATP led to the continuous dissolution of LLPS as indicated by the reduction of turbidity (Fig. 4a) and dissolution of droplets. At 1:750, all droplets have been completely dissolved (Fig. 4b) and no aggregation was observed even up to 1:1000. Most interestingly as shown in Fig. 4c, at 1:100, the majority of disappeared NMR peaks due to the presence of Tar32 has been restored. Nevertheless, some NMR peaks still have large shifts as compared to those in the free state or only in the presence of ATP. The addition of ATP to 1:500 resulted in the HSQC spectrum which is very similar to that only in the presence of ATP except for several peaks. Further addition of ATP to 1:1000 led to no further shifts.

In summary, the results together reveal that ATP and ssDNAs do interplay in modulating LLPS by competing for binding Arg/Lys. For the phase-separated state of Del-PLD with the hydrophobic region deleted which is thus only driven by the nucleic acid binding to Tar32, ATP is capable of completely dissolving it without inducing any aggregation. However, For the phase-separated states of WT-PLD in the presence of A6 or Tar32 as well as AllK-PLD in the presence of Tar32, which are maintained by both nucleic-acid-binding and oligomerization of the hydrophobic region, the competition between ATP and nucleic acids for binding Arg/Lys could all lead to exaggeration of LLPS into aggregation. Here we propose a potential mechanism: because ATP and nucleic acids are highly negatively charged, they can exert at least two strong electrostatic effects: namely site-/conformation-specific electrostatic interaction and non-specific screening effect. If ATP and nucleic acids are relatively tightly bound with Arg/Lys residues, site-/conformation-specific negative charges will be introduced onto PLD molecules. Consequently. the site-/conformation-specific repulsive electrostatic interaction will become dominant which not only contributes to dissolving LLPS, but also to preventing the aggregation triggered by hydrophobic interaction. By contrast, If they fail to be relatively tightly bound with Arg/Lys residues, the non-specific screening effect will become dominantly operating which will trigger aggregation driven by hydrophobic interaction, as we observed on AllA-PLD here as well as universally on the partially folded or disordered proteins with considerable exposure of hydrophobic patches, which are all soluble in unsalted water but become insoluble upon the introduction of salts even at very low concentrations^44^.

## Discussion

Although most, if not all, MLOs contain both nucleic acids and IDR-rich proteins, currently nucleic acids have been largely considered to exert non-specific electrostatic/salt effects onto LLPS. The present study, however, deciphers that ssDNAs are capable of driving and then dissolving LLPS of TDP-43 PLD with the capacity dependent on the length but not a sequence of ssDNAs. Very unexpectedly, extensive NMR characterization unambiguously provides the first residue-resolution evidence that ssDNAs achieve the modulation of LLPS mainly by the multivalent binding specifically to Arg/Lys residues with an affinity to Arg higher than that to Lys.

Together with recent NMR results with ATP^37,39,45,46^, a common mechanism is emerging for ATP and nucleic acids to modulate LLPS of IDRs by specific binding. Briefly, different from the binding to a well-folded protein to form the stable classic complex with a well-defined three-dimensional structure in which various types of residues are involved and a single atom variation of ATP/nucleic acids or proteins might dramatically alter the binding affinity, in forming the dynamic complex with IDRs which lack the defined conformation and thus are highly accessible to the bulk solvent, ATP/nucleic acids can only establish the NMR-detectable binding to Arg/Lys residues through electrostatic interactions between phosphate groups of ATP/nucleic acids and side chain cations of Arg/Lys as well as π–π/π–cation interactions between base aromatic rings and Arg/Lys side chains in which different bases have a highly similar affinity to Arg/Lys residues. Very recently, such interactions of ATP to Arg have also been uncovered by combining the semiempirical quantum mechanical method, mean-field theory, and molecular simulations^47^, which are fundamentally different from the non-specific electrostatic/salt effects and thus provide the specific capacity to ATP/nucleic acids for driving LLPS of Arg/Lys-containing IDRs. In this context, RNA and ssDNA are expected to bind Arg/Lys residues of IDRs with the same mechanism, because they only have two minor differences in their chemical structures.

On the other hand, nucleic acids differ from ATP in having multiple covalently linked nucleotides and consequently are capable of establishing multivalent binding to Arg/Lys to gain the length-dependent affinity. As such, nucleic acid could be approximately considered to be a polymer of ATP on the one hand. On the other hand, ATP can have a high freedom to orientate in binding while the nucleotides in nucleic acid have a highly restricted freedom to orientate in binding. thus resulting in different binding modes and excluding volumes. Chemically, ATP has a unique triphosphate group which appears to have a strong capacity in interacting with water molecules^35–39^ and the Arg side chain^47^, These differences may affect the competition between ATP and nucleic acid in binding Arg/Lys residues as well as their abilities to modulate LLPS. Intriguingly, although nucleic acid may acquire very high binding affinity to IDR by establishing multivalent but discrete binding to multiple Arg/Lys residues, such a high-affinity binding appears to be relatively vulnerable to be displaced by ATP as each of the discrete binding events can be simultaneously displaced by ATP.

As ATP and nucleic acids are highly negatively charged, they thus own the capacity to exert strong electrostatic effects onto LLPS and aggregation of IDRs, which, however, appear to be context-dependent. For example, ATP and nucleic acids impose at least dual effects on LLPS and aggregation of TDP-43 WT-PLD. Briefly, if in the presence of exceeding amounts, ATP or nucleic acids are able to become relatively tightly bound with its Arg/Lys residues, PLD molecules will become site-/conformation-specifically associated with multiple negative charges, whose repulsive electrostatic interaction acts not only to disrupt LLPS but also to prevent its irreversible aggregation. By contrast, if ATP or/and nucleic acids are unable to be bound with PLD, their non-specific screening effect will become dominantly operating to trigger aggregation driven by oligomerization of the unique hydrophobic region, as we universally observed on various aggregation-prone or even “insoluble” proteins with exposed hydrophobic patches, which are all soluble in salt-free water but become aggregated in salted solution^44^.

**Fig. 5:**
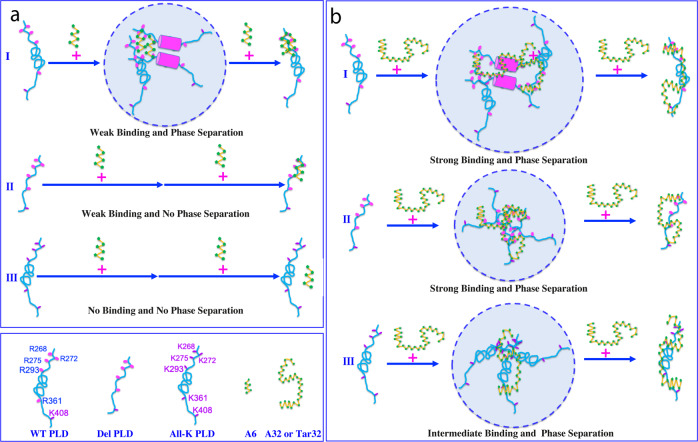
Speculative mechanisms for three ssDNAs to three PLDs to modulate LLPS. **a** Interactions of A6 to three PLDs. (I) For WT-PLD, A6 multivalently binds its clustered Arg268, Arg272, and Arg275 of several WR-PLD molecules to form large and dynamic A6-PLD complexes manifesting as liquid-like droplets, which is coupled with the intrinsic driving force via the oligomerization-induced formation of helices (purple cylinder) over the hydrophobic region. However, with further addition of an exceeding amount A6, LLPS is disrupted. (II) For Del-PLD, A6 is still able to multivalently bind its clustered Arg268, Arg272, and Arg275 but is no longer able to drive LLPS due to the deletion of the hydrophobic region. (III) For AllK-PLD, A6 is no longer able to tightly bind its residues as well as unable to drive LLPS due to the low binding affinity of Lys residues with nucleic acids. **b** Interactions of Tar32/A32 to three PLDs. (I) For WT-PLD, Tar32/A32 multivalently binds not only the clustered Arg268, Arg272, and Arg275, but also Arg293, Arg361, and Lys408 to form large and dynamic Tar32/A32–PLD complexes manifesting as liquid-like droplets, which is coupled with the intrinsic driving force via the oligomerization-induced formation of helices (purple cylinder) over the hydrophobic region. With the further addition of an exceeding amount Tar32/A32, LLPS is disrupted. (II) For Del-PLD, the multivalent binding of Tar32/A32 to the clustered Arg268, Arg272, and Arg275, as well as Arg293, Arg361, and Lys408 are sufficient to form large and dynamic Tar32/A32–PLD complexes manifesting as liquid-like droplets despite the lack of the intrinsic driving force. With the further addition of an exceeding amount Tar32/A32, LLPS is disrupted. (III) For AllK-PLD, the multivalent binding of Tar32/A32 to Lys268, Lys272, and Lys275, as well as Lys293, Lys361, and Lys408 are sufficient to form large and dynamic Tar32/A32–PLD complexes manifesting as liquid-like droplets despite the low binding affinity of Lys residues with nucleic acids. With the further addition of an exceeding amount Tar32/A32, LLPS is disrupted.

In this framework, the mechanisms for three ssDNAs to interact with three PLDs can be formulated. As illustrated in Fig. 5, the short A6 appears to be only able to maximally achieve multivalent binding to the clustered Arg268, Arg272, and Arg275 and gains a relatively weak affinity. Therefore, only in the presence of the intrinsic driving force from the oligomerization of the hydrophobic region, A6 is able to drive LLPS of WT-PLD to form dynamically and multivalently cross-linked A6-PLD complex which manifests as liquid droplets. Further addition of A6 to an exceeding amount will lead to the formation of A6-bound PLD which is highly negatively charged. Consequently, the droplets will be disrupted as well as the aggregation of WT-PLD triggered by the oligomerization of the hydrophobic region is inhibited by the repulsive electrostatic interaction (I of Fig. 5a). In this regard, despite binding to the same Arg residues of Del-PLD with a similar affinity, A6 can no longer drive its LLPS because of the deletion of the intrinsic driving force (II of Fig. 5a). Furthermore, due to the much weaker binding affinity between the base ring and Lys side chain, A6 has almost no detectable binding to Lys of AllK-PLD as well as is unable to drive its LLPS (III of Fig. 5a). By contrast, in addition to binding the clustered Arg268, Arg272 and Arg275, the long Tar32/A32 appears to further bind Arg293, Arg361, and Lys408 and consequently acquire the affinity much high than that of A6. As such, for WT-PLD, Tar32/A32 drives LLPS by coupling both nucleic-acid-binding and intrinsic driving force to form the dynamic and multivalent Tar32/A32–PLD complex also manifesting as liquid droplets. Similarly, further addition of Tar32/A32 will also lead to the formation of highly negatively charged Tar32/A32-bound PLD. Consequently, the droplets will be disrupted and the aggregation is inhibited (I of Fig. 5b). Importantly, due to the high binding affinity, Tar32/A32 is still able to drive and then dissolve LLPS of the 117-residue Del-PLD even with the intrinsic driving force deleted (II of Fig. 5b). Furthermore, although the individual binding affinity of each base ring to Lys is much weaker than that to Arg, the overall affinity resulting from the multivalent binding of long Tar32/A32 to multiple Lys residues of AllK-PLD is still sufficiently high to drive and then dissolve its LLPS (III of Fig. 5b).

These results together not only unveil the first nucleic-acid-driven mechanism for LLPS of TDP-43 PLD; but also decode a general principle that not just TDP-43 PLD, all Arg/Lys-containing IDRs, which are not limited to those rich in RG-/RGG-motifs as the C-terminal domain of FUS^38^, can act as ATP and nucleic-acid-binding domains. Most strikingly, the multivalent and specific binding of their Arg/Lys residues with nucleic acids of a sufficient length is sufficient to drive and dissolve their LLPS without the need for any other driving force. Furthermore, ATP and nucleic acids universally interplay to modulate LLPS by competing for binding Arg/Lys of IDRs. However, for TDP-43 WT-PLD containing the hydrophobic region, whose oligomerization also serves to drive LLPS, its LLPS is prone to exaggeration into irreversible aggregation, particularly when ATP and nucleic acids co-exist to compete for binding Arg/Lys. Their competition may lead to a reduction of the binding affinity of both of them. Consequently, the negative charges of ATP or/and nuclei acids site-/conformation-specifically associated with Arg/Lys of PLD will become dissociated to some degree. Under such a circumstance, the repulsive electrostatic interaction among PLD molecules will be reduced while the non-specific screening effects imposed by ATP and nucleic acids will become operating dominantly to enhance oligomerization of the hydrophobic region, which eventually results in exaggerating LLPS into the irreversible aggregation.

## Conclusion

In contrast to the widespread belief, the present study decodes that mainly by multivalently and specifically binding Arg/Lys residues, nucleic acids act to drive and then dissolve LLPS of TDP-43 PLD with the length-dependent capacity. Although for the special case of TDP-43 WT-PLD, the nucleic-acid-binding drives its LLPS by coupling the intrinsic driving force, in the general context the multivalent nucleic-acid-binding itself is sufficient to drive and then dissolve LLPS of Arg-/Lys-containing IDRs without the need of any other driving force. Most strikingly, ATP and nucleic acids have been decrypted to share the common mechanism in biding Arg/Lys of IDRs. Consequently, not just TDP-43 PLD, all Arg/Lys-containing IDRs are cryptic domains for binding ATP and nucleic acids and may thus phase separate upon multivalently binding nucleic acids.

Most strikingly, ATP and nucleic acids universally interplay in modulating LLPS by competing for binding Arg-/Lys. This discovery bears immediate implications for our understanding of the homeostasis of fundamental cellular processes. So far, even RG-/RGG-rich IDRs have been identified in >1700 human proteins^48–52^. Generally, it was estimated that the average frequency of Arg found in eukaryotic protein is ~5.6% while that of Lys is ~6.2^53^. As such, IDRs are anticipated to contain multiple Arg/Lys whose number is comparable to or even higher than that of TDP-43 PLD unless their frequency of Arg/Lys is strongly biased and lower than the average frequency. Indeed, for example, the 40-residue intrinsically disordered Nogo-40, which has no known function to bind ATP and nucleic acids to undergo LLPS, contains two Arg and three Lys residues^54^. In this context, most, if not all, IDRs, which account for about the half of human proteome, are capable of multivalently binding nucleic acids to undergo LLPS.

Intriguingly, however, only a very limited number of MLOs have been identified in cells. One possible mechanism underlying this puzzling observation is that ATP with high cellular concentrations might play a previously-unrecognized role in inhibiting the LLPS of most Arg/Lys-containing IDRs. It appears that even for the formed IDR–nucleic-acid complexes and MLOs such as SGs, ATP is still essential to maintain their functional dynamics or reversibility as well as to prevent their exaggeration into irreversible aggregation, which are associated with an increasing spectrum of human diseases including all neurodegenerative diseases. Therefore, the unregulated introduction of exogenous nucleic acids into cells may lead to disastrous perturbations to the homeostasis of the interactions among ATP, nucleic acids, and proteins, such as the formation of abnormal IDR-nucleic-acid complexes, thus triggering various diseases. Indeed, even the abnormally high affinity of Arg/Lys-containing IDRs to nucleic acids appears to provoke lethal cytotoxicity. For example, the ALS-causing C9orf72 dipeptide repeats extremely rich in Arg have been recently shown to have an extremely high affinity to nucleic acids and consequently to generally displace RNA/DNA-binding proteins from binding mRNA in chromatin, which impairs any processes involving nucleic acids^55^.

## Methods

### Preparation of recombinant WT and mutated TDP-43 PLD proteins

Here, we used our previously cloned DNA constructs in a modified vector without any tag which include those encoding TDP-43 WT-PLD over residues 265–414, Del-PLD with residues 311–343 deleted, and AllK-PLD with all five Arg replaced by Lys^37^. All three recombinant TDP-43 PLD proteins were highly expressed in *E. coli* BL21 cells and were found in inclusions. Consequently, they were purified by the previously established protocols in other and our labs. Briefly, the recombinant proteins were solubilized with the buffer with 8 M urea, and the reverse phase (RP)-HPLC purification was used to obtain highly pure proteins with the impurities including liquid, ions, and nucleic acids removed^37^. Isotope-labeled proteins for NMR studies were prepared by the same procedures except that the bacteria were grown in M9 medium with the addition of (^15^NH_4_)_2_SO_4_ for ^15^N-labeling. The protein concentration was determined by the UV spectroscopic method in the presence of 8 M urea, under which the extinct coefficient at 280 nm of a protein can be calculated by adding up the contribution of Trp, Tyr, and Cys residues^37,56^.

ATP was purchased from SigmaAldrich with the same catalog numbers as previously reported^37^. Three synthetic ssDNAs were purchased from a local company^33^. Proteins, ssDNA, and ATP samples were all prepared in 10 mM sodium phosphate buffer and MgCl_2_ at the equal molar concentration to ATP was added for stabilization by forming the ATP–Mg complex^37^. The final solution pH values were checked by pH meter and the small variations were adjusted with aliquots of very diluted NaOH or HCl^37^.

### Differential interference contrast (DIC) microscopy and turbidity measurement

The formation of liquid droplets was imaged at 25 °C on 50 µl of different TDP-43 PLD samples at 15 μM in 10 mM sodium phosphate in the absence and in the presence of ssDNAs at different molar ratios by differential interference contrast (DIC) microscopy (OLYMPUS IX73 Inverted Microscope System with OLYMPUS DP74 Color Camera)^37^. The turbidity measurement and DIC imaging were performed after 15 min of the sample preparation. The turbidity was measured three times at a wavelength of 600 nm and reported as Average + SD.

### NMR characterizations

All NMR experiments were acquired at 25 °C on an 800 MHz Bruker Avance spectrometer equipped with pulse field gradient units and a shielded cryoprobe^37^. To have the enhancing effect of the cryoprobe for NMR signal sensitivity, which is essential for NMR HSQC titration experiments at such a low protein concentration (15 µM), NMR samples had to be prepared in 10 mM sodium phosphate buffer, while pH value was optimized to 5.5 as many HSQC peaks of TDP-43 PLD disappeared at higher pH values due to the enhanced exchange with bulk solvent and/or dynamic association.

For NMR titration studies of the interactions between TDP-43 WT/mutated PLD proteins and ATP and ssDNAs, one-dimensional proton and two-dimensional ^1^H–^15^N NMR HSQC spectra were collected on ^15^N-labeled samples at a protein concentration of 15 µM in 10 mM sodium phosphate buffer (pH 5.5) at 25 °C in the absence and in the presence of ssDNAs or ATP at different molar ratios.

NMR data were processed by NMRPipe^57^ and analyzed by NMRView^58^. To calculate chemical shift difference (CSD) induced by interacting with ssDNAs, HSQC spectra were superimposed, and subsequently, the shifted peaks were identified, which were further assigned to the corresponding residues of TDP-43 PLD with the NMR resonance assignments previously achieved by us and other groups^37^. The degree of the perturbation was reported by an integrated index calculated by the following formula^37,40^:

1 $${{{\rm{CSD}}}}\,({{{\rm{ppm}}}})=(({\Delta }^{1}{{{{\rm{H}}}}})^{2}+({\Delta }^{15}{{{{\rm{N}}}}})^{2}{/4})^{1/2}$$

The residues with CSD values > Average + STD are defined as significantly perturbed residues.

### Statistics and reproducibility

For NMR and DIC experiments, the exploratory experiments of TDP-43 PLD and its mutants titrated with ATP and three ssDNAs at different concentrations were first conducted to identify the optimized concentration ranges. Subsequently, the final DIC and HSQC titrations were performed once with the optimized points of ATP and three ssDNAs concentrations.

### Reporting summary

Further information on research design is available in the Nature Portfolio Reporting Summary linked to this article.

## Supplementary information

Supplementary Information

Description of Additional Supplementary Files

Supplementary Data 1

Reporting Summary

## Data Availability

The data supporting the findings of this study are available within the paper and Supplementary Data 1. All other data are available from the corresponding author upon reasonable request.
