# Supplementary material for: Arg/Lys-containing IDRs are cryptic binding domains for ATP and nucleic acids that interplay to modulate LLPS
Source: Commun Biol. 2022 Dec 1;5:1315. doi: 10.1038/s42003-022-04293-w (PMC9712531; doi:10.1038/s42003-022-04293-w)
Supplement: Supplementary file 1 — Supplementary Information [file 42003_2022_4293_MOESM1_ESM.pdf]

Arg/Lys-containing IDRs are cryptic binding domains for ATP and nucleic acids that interplay to modulate LLPS

Dang Mei, Tongyang Li, Shibo Zhou and Jianxing Song*
Department of Biological Sciences, Faculty of Science; National University of Singapore; 10 Kent Ridge Crescent, Singapore 119260

Short title: Mechanism of nucleic-acid-driven LLPS of TDP-43 PLD

*** Corresponding author; Email:** dbssjx@nus.edu.sg.

Supplementary Figures 1-16

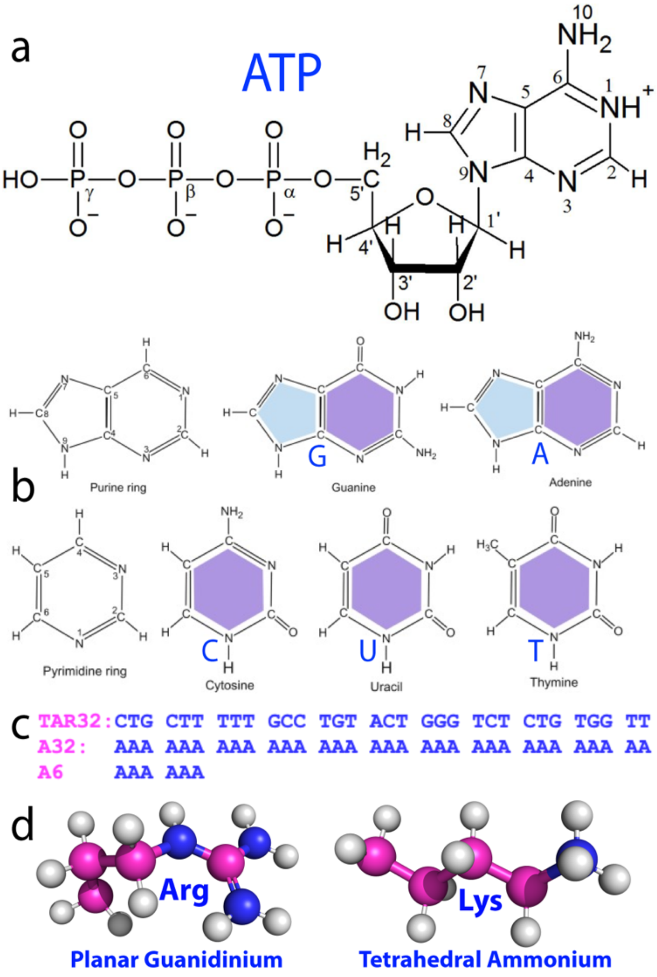

Supplementary Figure 1. Chemical structures and sequences.
Chemical structures of ATP (a) and nitrogenous bases in DNA and RNA (b). (c) Sequences of ssDNA Tar32, A32 and A6. (d) Stick-and-ball models of the side chains of Arg and Lys residues showing the planar guanidinium cation of arginine and tetrahedral ammonium cation of lysine.

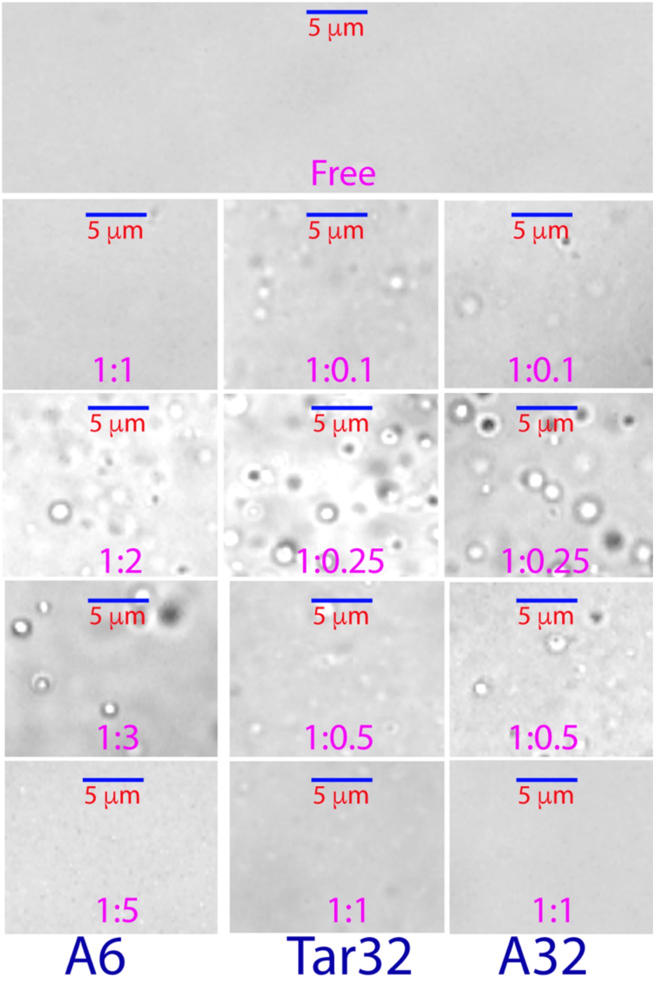

Supplementary Figure 2. Three ssDNAs modulate LLPS of TDP-43 WT-PLD.
DIC images of WT-PLD in the presence of A6, Tar32 and A32 at different molar ratios.

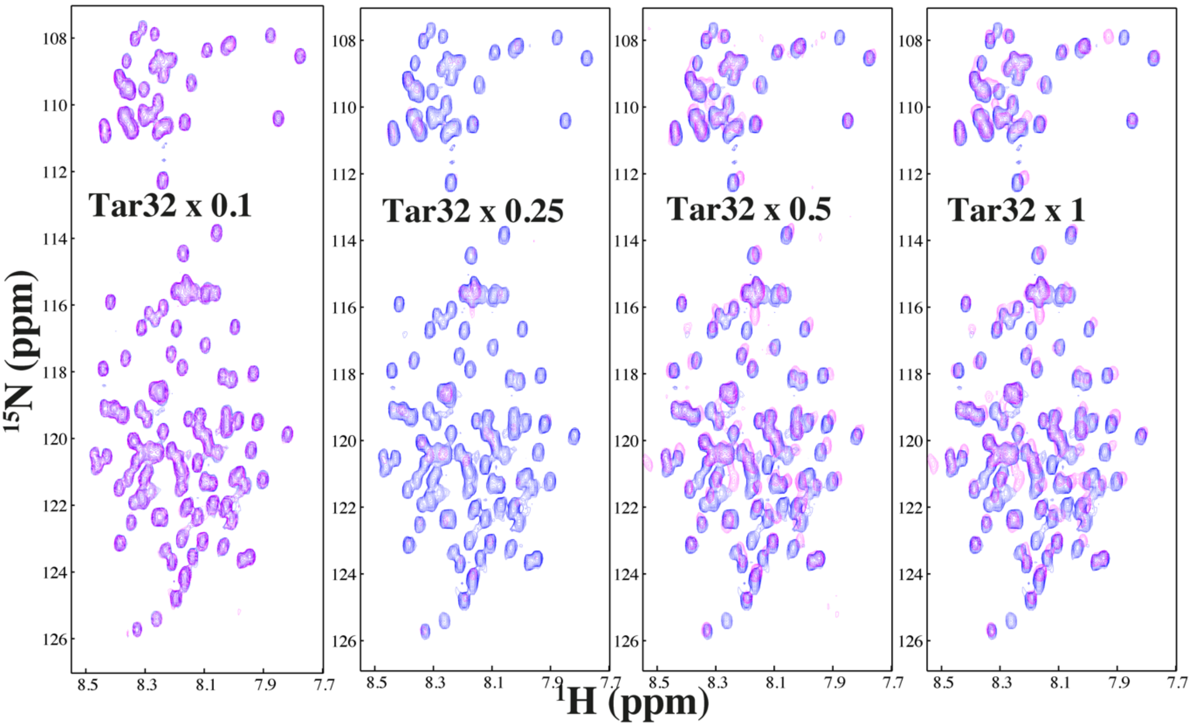

Supplementary Figure 3. NMR characterization of the binding of WT-PLD to Tar32. ^{1}H-^{15}N NMR HSQC spectra of ^{15}N-labeled WT-PLD in the absence (blue) and in the presence of Tar32 at different molar ratios (purple).

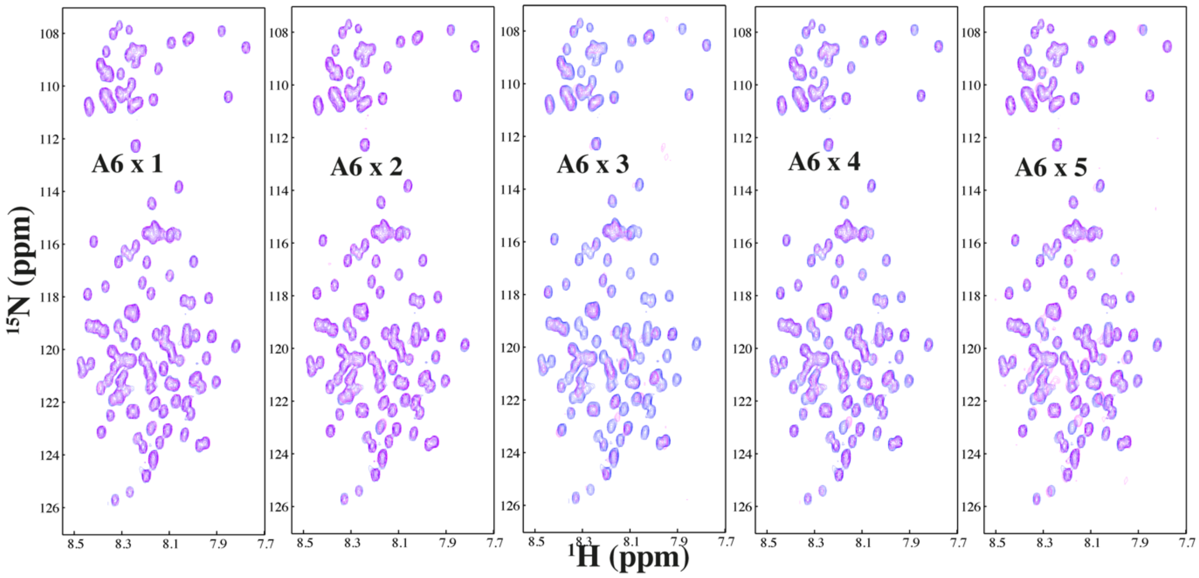

Supplementary Figure 4. NMR characterization of the binding of WT-PLD to A6. HSQC spectra of WT-PLD in the absence (blue) and in the presence of A6 at different molar ratios (purple).

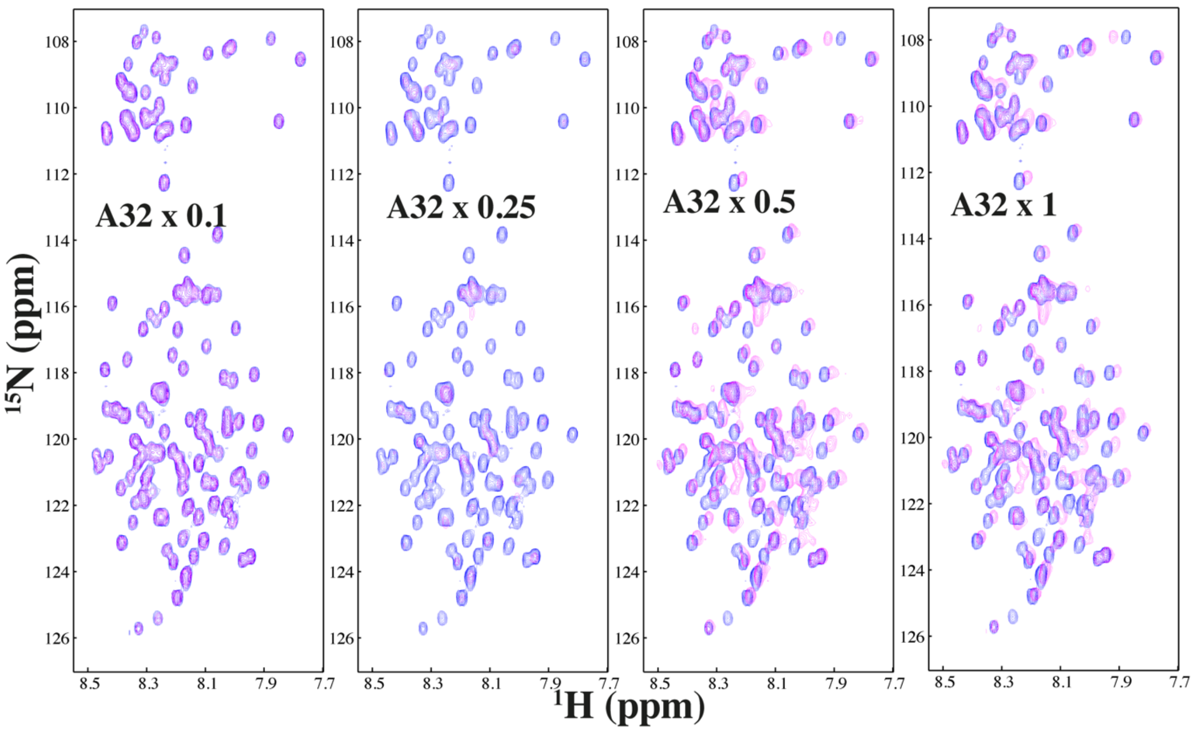

Supplementary Figure 5. NMR characterization of the binding of WT-PLD to A32. HSQC spectra of WT-PLD in the absence (blue) and in the presence of A32 at different molar ratios (purple).

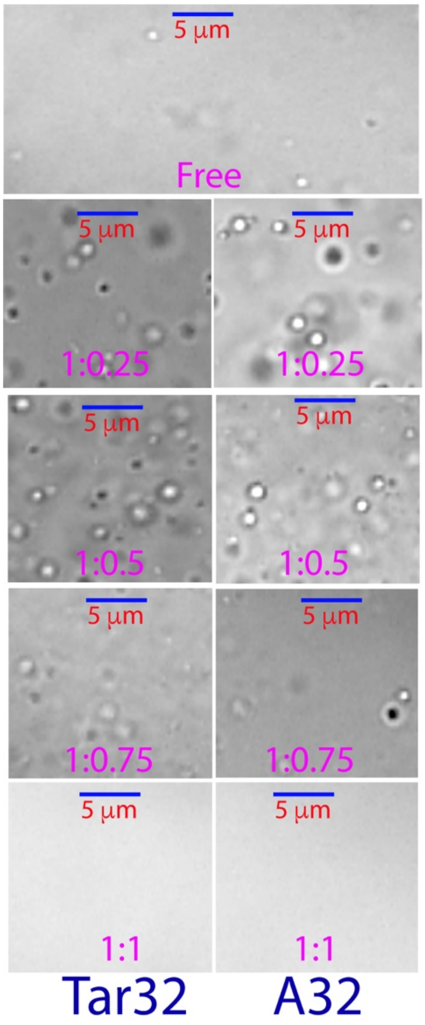

Supplementary Figure 6. Two ssDNAs modulate LLPS of TDP-43 AllK-PLD.
DIC images of AllK-PLD in the presence of Tar32 and A32 at different molar ratios.

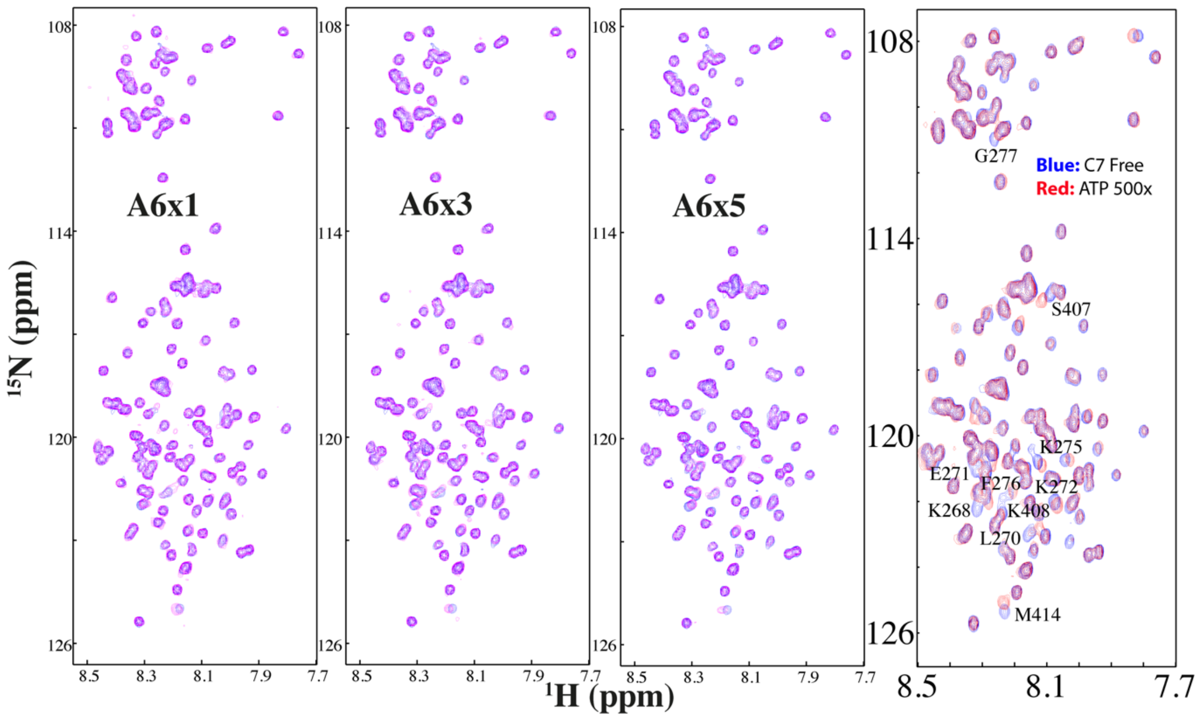

Supplementary Figure 7. NMR characterization of the binding of AllK-PLD to A6. HSQC spectra of AllK-PLD in the absence (blue) and in the presence of A6 at different molar ratios (purple).

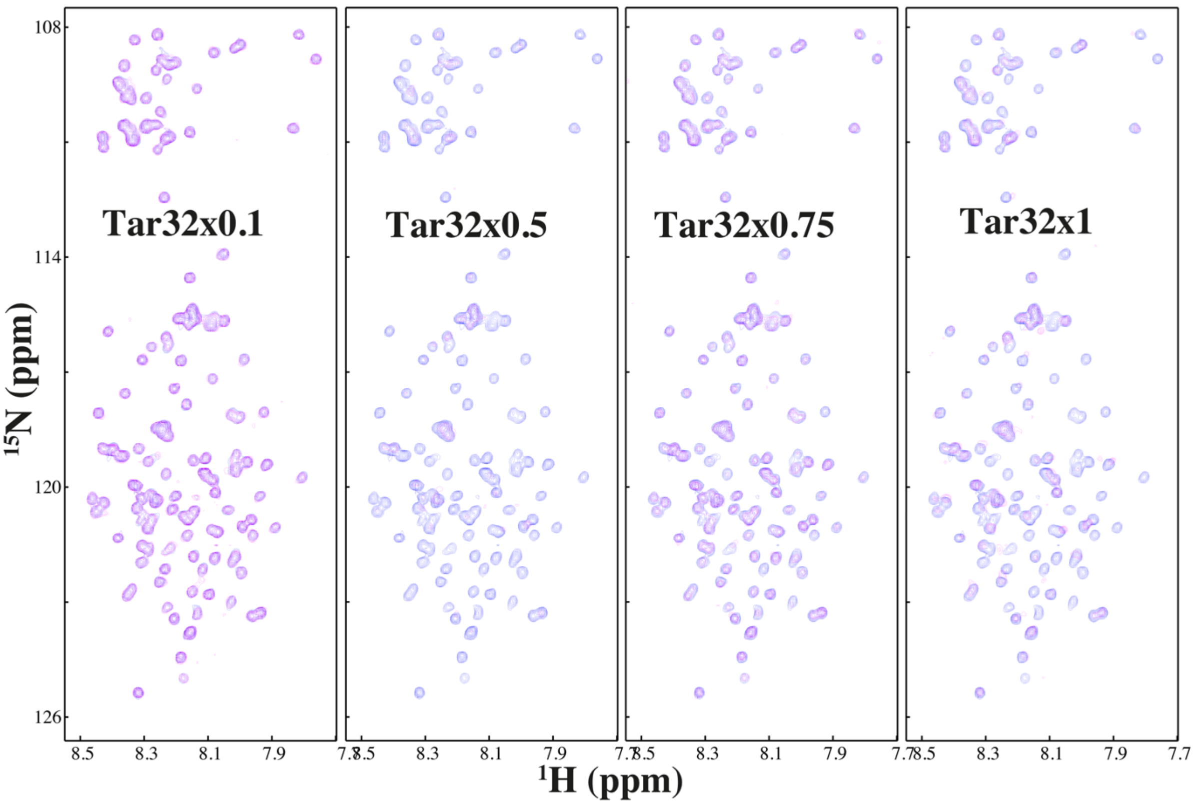

Supplementary Figure 8. NMR characterization of the binding of AllK-PLD to Tar32. HSQC spectra of AllK-PLD in the absence (blue) and in the presence of Tar32 at different molar ratios (purple).

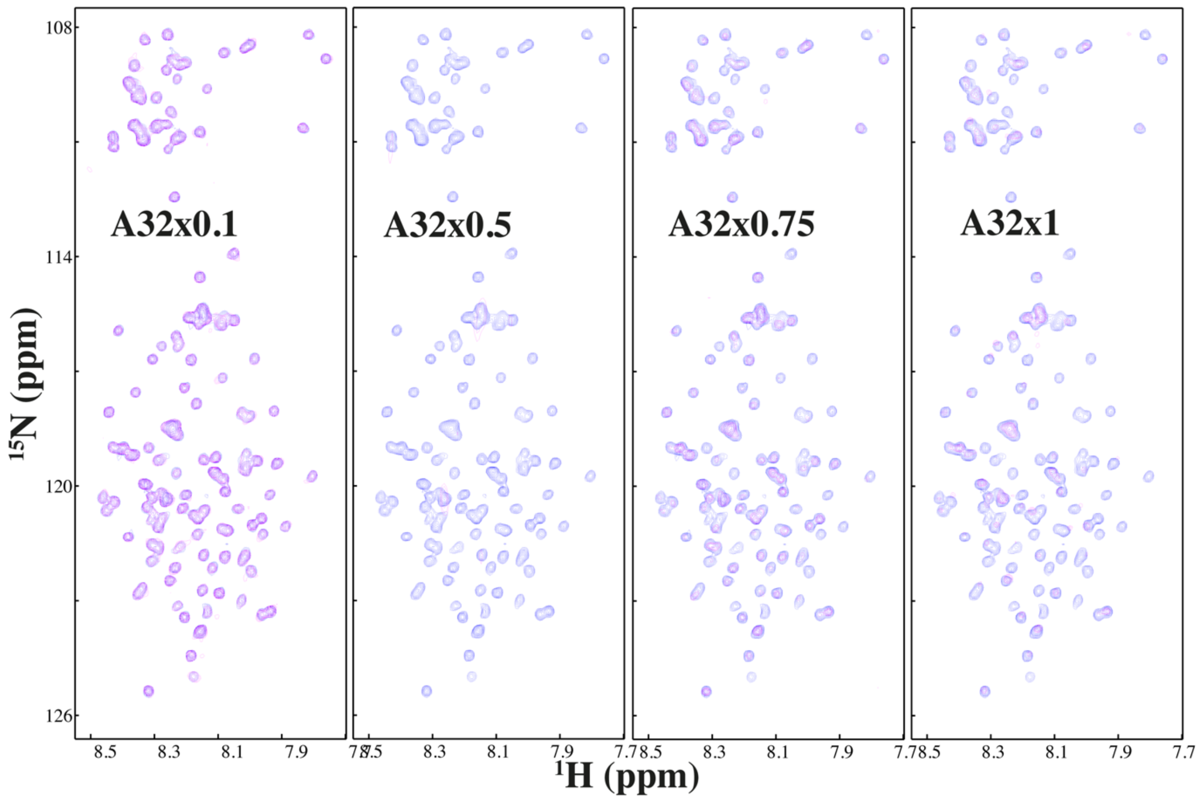

Supplementary Figure 9. NMR characterization of the binding of AllK-PLD to A32. HSQC spectra of AllK-PLD in the absence (blue) and in the presence of A32 at different molar ratios (purple).

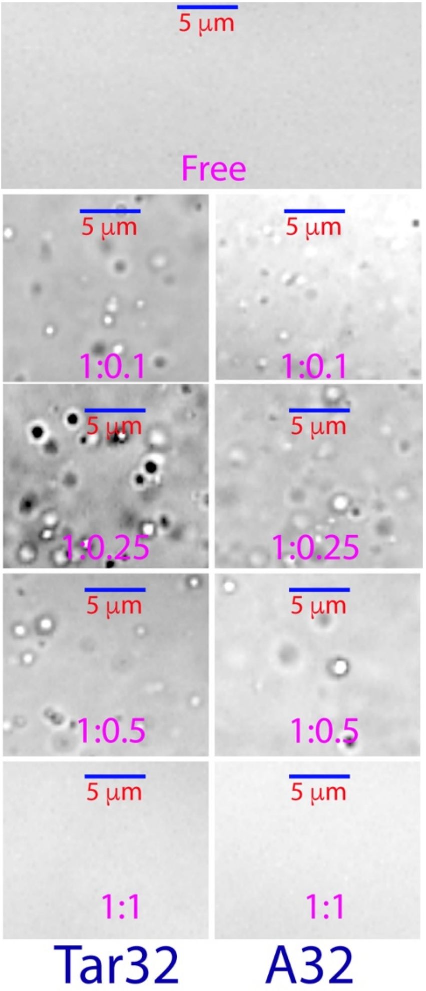

Supplementary Figure 10. Two ssDNAs modulate LLPS of TDP-43 Del-PLD.
DIC images of Del-PLD in the presence of Tar32 and A32 at different molar ratios.

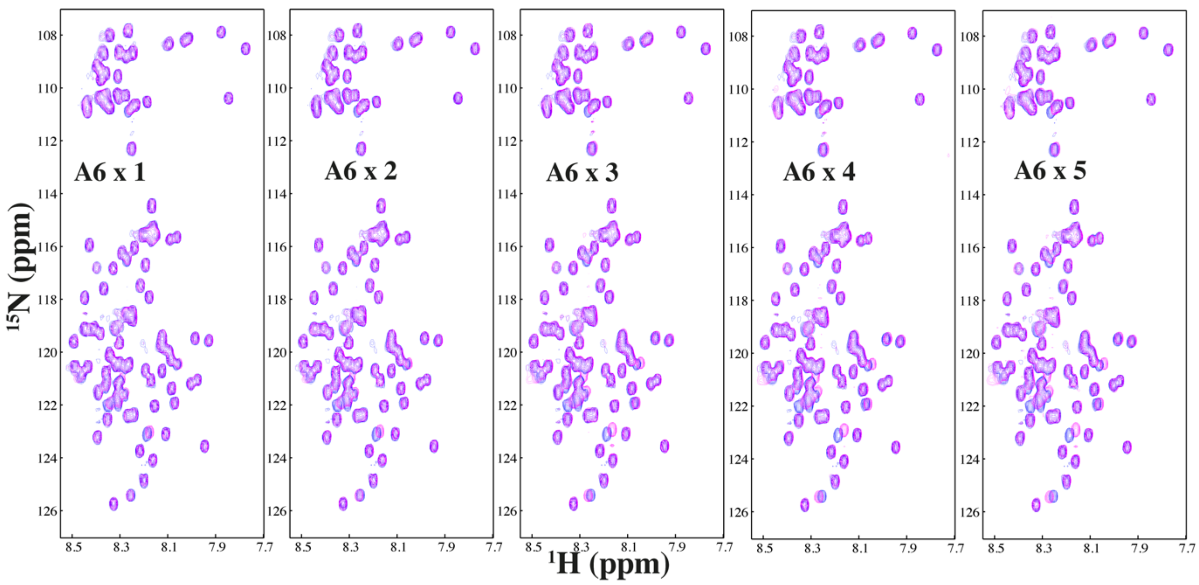

Supplementary Figure 11. NMR characterization of the binding of Del-PLD to A6. HSQC spectra of Del-PLD in the absence (blue) and in the presence of A6 at different molar ratios (purple).

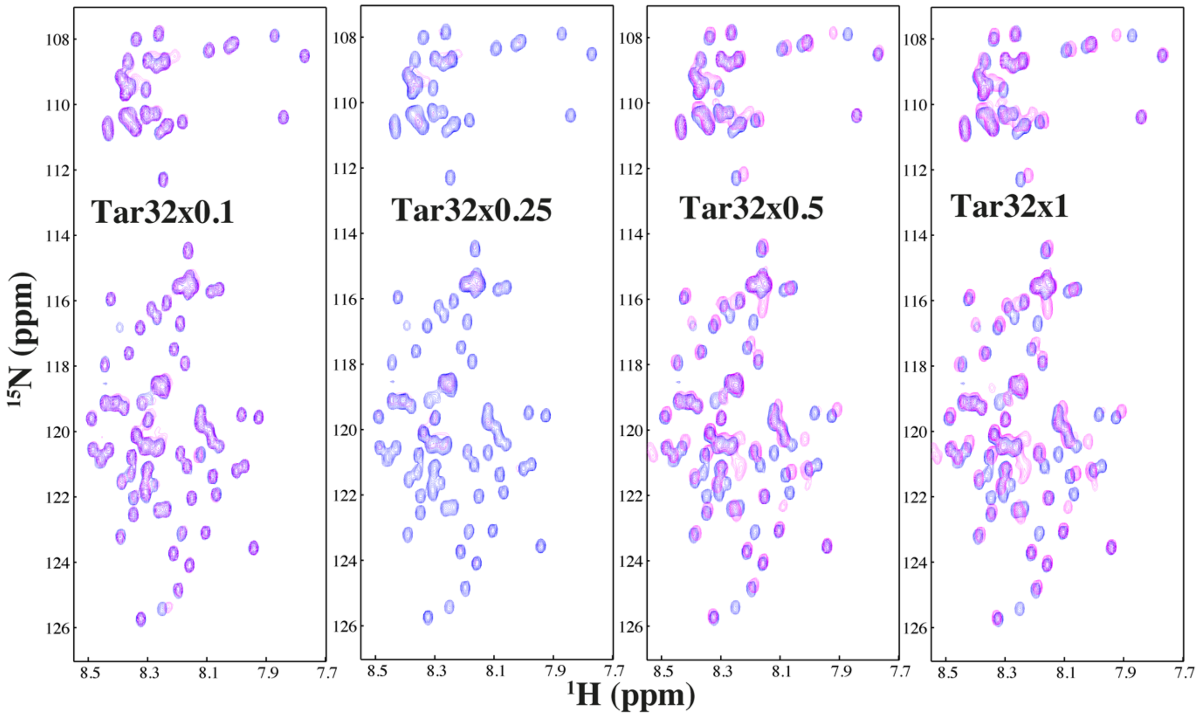

Supplementary Figure 12. NMR characterization of the binding of Del-PLD to Tar32. HSQC spectra of Del-PLD in the absence (blue) and in the presence of Tar32 at different molar ratios (purple).

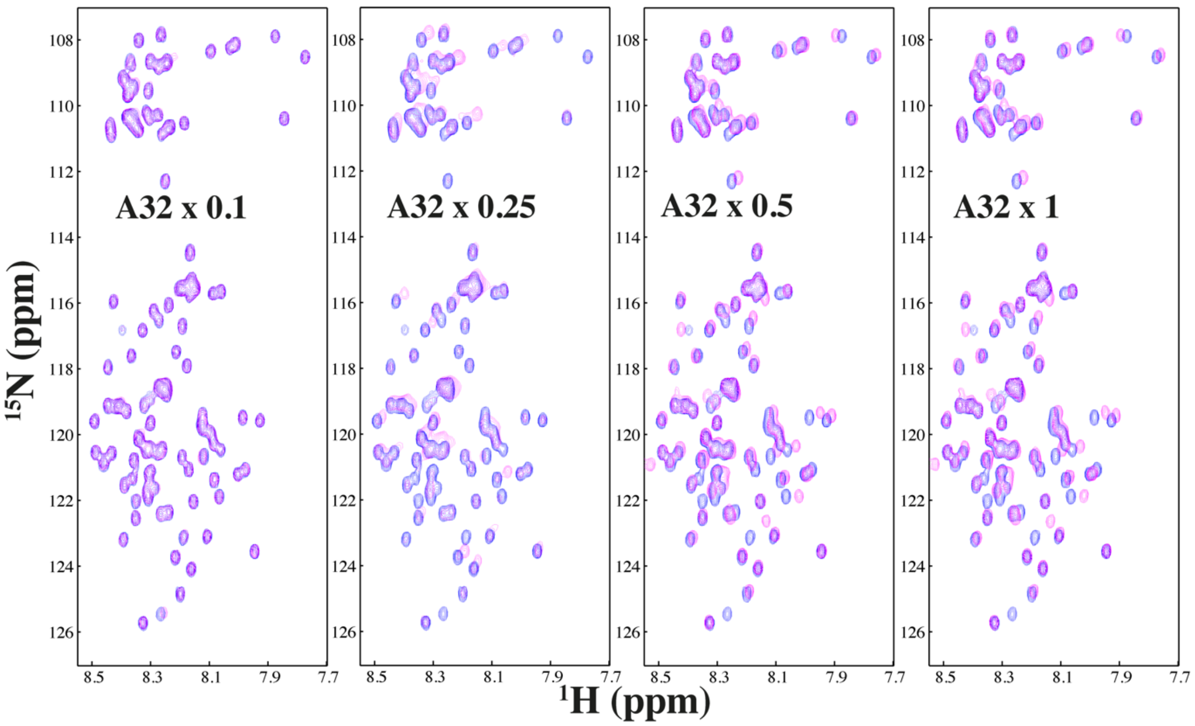

Supplementary Figure 13. NMR characterization of the binding of Del-PLD to A32. HSQC spectra of Del-PLD in the absence (blue) and in the presence of A32 at different molar ratios (purple).

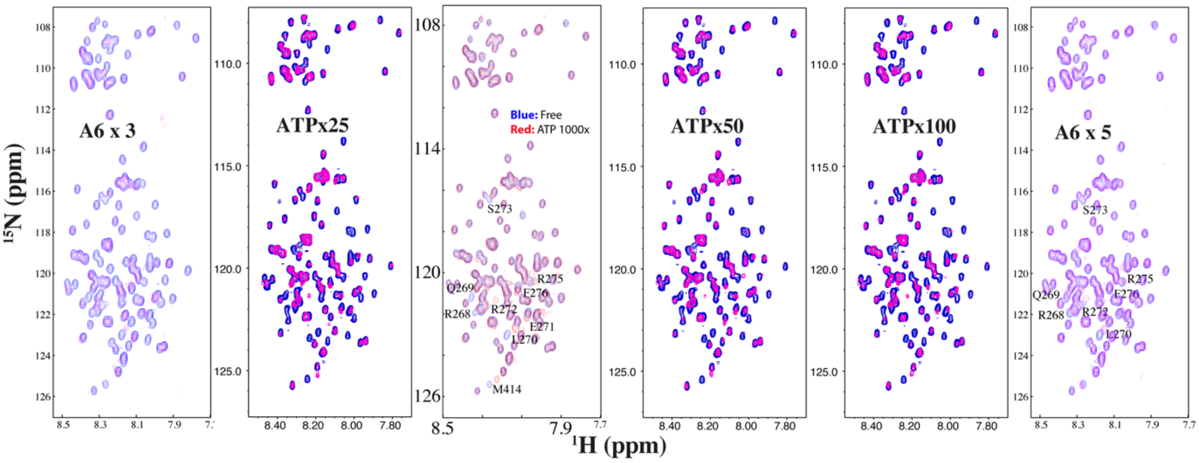

Supplementary Figure 14. NMR characterization of ATP titrations on A6-maintained LLPS of WT-PLD.

HSQC spectra of WT-PLD in the absence (blue) and in the presence of A6 at 1:3 (purple), and with further addition of ATP at different ratios.

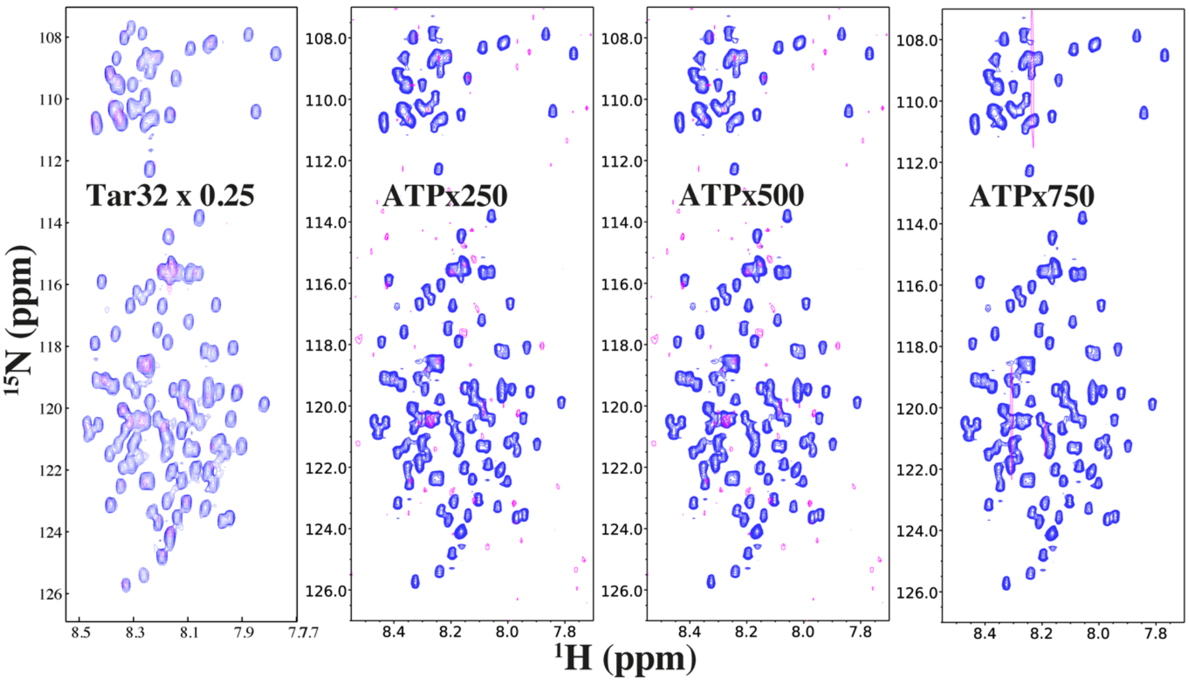

Supplementary Figure 15. NMR characterization of ATP titrations on Tar32-maintained LLPS of WT-PLD.

HSQC spectra of WT-PLD in the absence (blue) and in the presence of Tar32 at 1:0.25 (purple), and with further addition of ATP at different ratios.

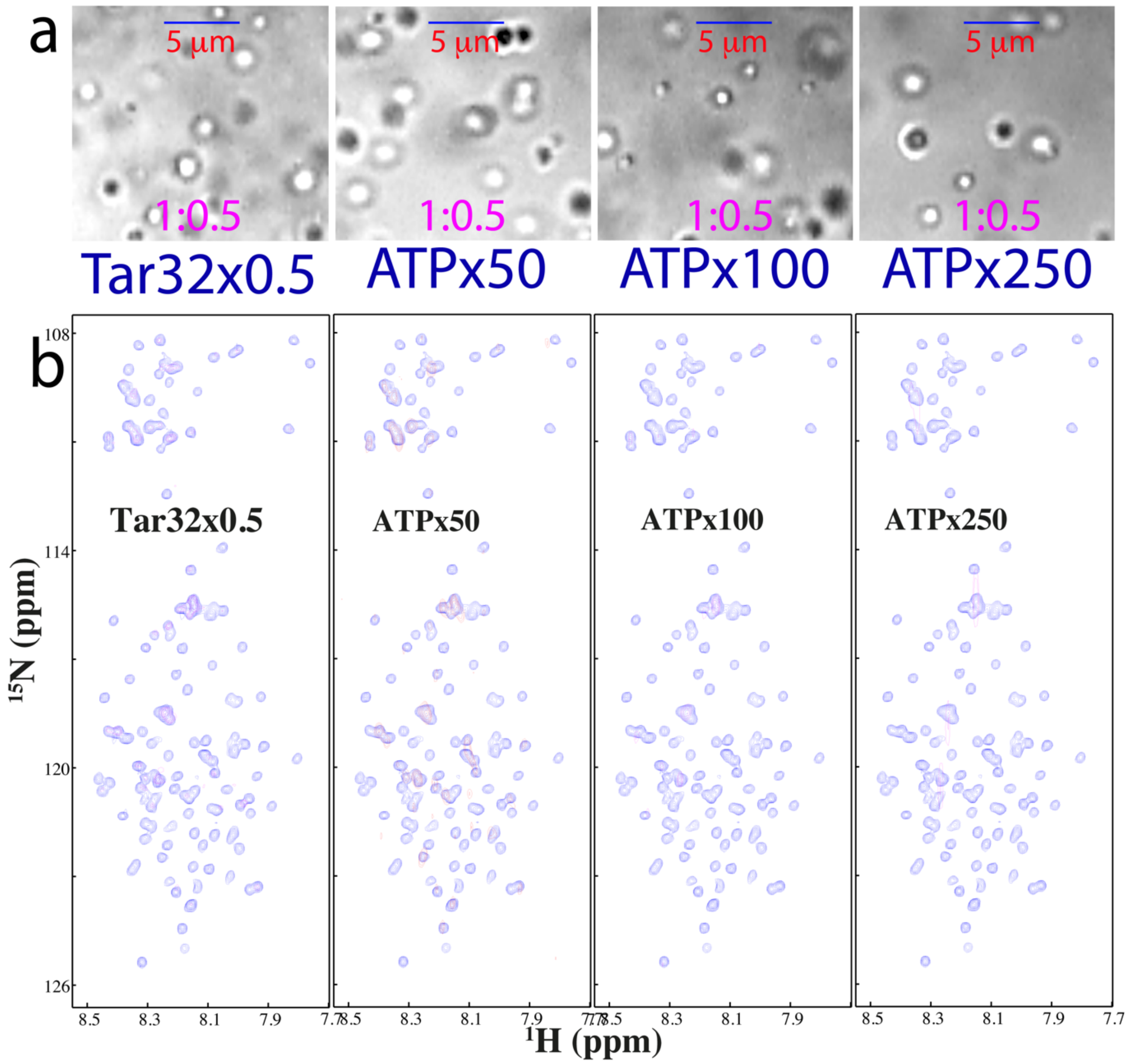

Supplementary Figure 16. NMR characterization of ATP titrations on Tar32-maintained LLPS of AllK-PLD.
(a) DIC images of AllK-PLD in the presence of Tar32 at 0.5 and with further titrations of ATP at different molar ratios. (b) HSQC spectra of AllK-PLD in the absence (blue) and in the presence of Tar32 at 1:0.5 (purple), and with further titrations of ATP at different ratios.
