# Supplementary material for: Arg/Lys-containing IDRs are cryptic binding domains for ATP and nucleic acids that interplay to modulate LLPS
Source: Commun Biol. 2022 Dec 1;5:1315. doi: 10.1038/s42003-022-04293-w (PMC9712531; doi:10.1038/s42003-022-04293-w)
Supplement: Supplementary file 2 — Description of Additional Supplementary Files [file 42003_2022_4293_MOESM2_ESM.pdf]

Description of Additional Supplementary Files

File name: Supplementary Data 1

Description: an excel file which contains all turbidity and NMR data used for preparing main figures.
